# Health information system (HIS) assessments as the starting point for HIS strengthening

**DOI:** 10.1093/eurpub/ckac129.133

**Published:** 2022-10-25

**Authors:** David Ortiz

**Affiliations:** Data and Digital Health Unit, WHO Regional Office for Europe, Copenhagen, Denmark

## Abstract

One of main the HIS strengthening tools applied by WHO-Euro is the ‘Support tool to assess health information systems and develop and strengthen health information strategies’, which was first published in 2015 and updated in 2021. The support tool comprises two main parts: it provides guidance firstly for performing an overall assessment of the full HIS, and secondly, for the subsequent development of an HIS strategy. The common mode of application of this tool is an external HIS assessment by a WHO team and a subsequent country-led process of HIS strategy development. So far, experience in the field has been gained mainly with the assessment part of the tool, which has been applied in 15 MS. The assessment process starts with a preparatory desk review, followed by a country visit during which semi-structured interviews are held with key HIS stakeholders. The guidance for the interviews consists of a core module and several add-on modules. The aim of the core module is to provide an overview of the functioning of the entire national HIS, while the add-on modules shed more light on specific parts or functions of the HIS, such as noncommunicable diseases monitoring, human resources for health or health data governance. The core module forms the basis of the HIS assessments, and one or several add-on modules can be added to it, according to the needs of the assessed country. The country visit is finalised with a multi-stakeholder debriefing. After the visit, a report is written with a summary of the situation in the country including an HIS maturity score, an analysis of strengths, weaknesses, opportunities, and threats (SWOT), and recommendations for improvement for the short, medium, and long term. In this presentation, we will explain the methodology of the assessment tool, and reflect on its added value as well as challenges encountered when applying it in practice.

